# Two intestinal microbiota-derived metabolites, deoxycholic acid and butyrate, synergize to enhance host defense peptide synthesis and alleviate necrotic enteritis

**DOI:** 10.1186/s40104-024-00995-9

**Published:** 2024-03-02

**Authors:** Dohyung M. Kim, Jing Liu, Melanie A. Whitmore, Isabel Tobin, Zijun Zhao, Guolong Zhang

**Affiliations:** Department of Animal and Food Sciences, Oklahoma State University, Stillwater, OK USA

**Keywords:** Bile acids, Host defense peptides, Metabolites, Microbiome, Necrotic enteritis, Short-chain fatty acids

## Abstract

**Background:**

Necrotic enteritis (NE) is a major enteric disease in poultry, yet effective mitigation strategies remain elusive. Deoxycholic acid (DCA) and butyrate, two major metabolites derived from the intestinal microbiota, have independently been shown to induce host defense peptide (HDP) synthesis. However, the potential synergy between these two compounds remains unexplored.

**Methods:**

To investigate the possible synergistic effect between DCA and butyrate in regulating HDP synthesis and barrier function, we treated chicken HD11 macrophage cells and jejunal explants with DCA and sodium butyrate (NaB), either individually or in combination, for 24 h. Subsequently, we performed RNA isolation and reverse transcription-quantitative PCR to analyze HDP genes as well as the major genes associated with barrier function. To further determine the synergy between DCA and NaB in enhancing NE resistance, we conducted two independent trials with Cobb broiler chicks. In each trial, the diet was supplemented with DCA or NaB on the day-of-hatch, followed by NE induction through sequential challenges with *Eimeria maxima* and *Clostridium perfringens* on d 10 and 14, respectively. We recorded animal mortality after infection and assessed intestinal lesions on d 17. The impact of DCA and NaB on the microbiota in the ileum and cecum was evaluated through bacterial 16S rRNA gene sequencing.

**Results:**

We found that the combination of DCA and NaB synergistically induced multiple HDP genes in both chicken HD11 cells and jejunal explants. Additionally, the gene for claudin-1, a major tight junction protein, also exhibited synergistic induction in response to DCA and NaB. Furthermore, dietary supplementation with a combination of 0.75 g/kg DCA and 1 g/kg NaB led to a significant improvement in animal survival and a reduction in intestinal lesions compared to either compound alone in a chicken model of NE. Notably, the cecal microbiota of NE-infected chickens showed a marked decrease in SCFA-producing bacteria such as *Bacteroides, Faecalibacterium*, and *Cuneatibacter*, with lactobacilli becoming the most dominant species. However, supplementation with DCA and NaB largely restored the intestinal microbiota to healthy levels.

**Conclusions:**

DCA synergizes with NaB to induce HDP and claudin-1 expression and enhance NE resistance, with potential for further development as cost-effective antibiotic alternatives.

**Supplementary Information:**

The online version contains supplementary material available at 10.1186/s40104-024-00995-9.

## Introduction

Necrotic enteritis (NE) not only causes a $6 billion annual economic loss to the global poultry industry, but also poses a serious human health risk due to the zoonotic potential of its etiological agent, *Clostridium perfringens* [1, 2]. There are currently no effective prophylactic or therapeutic measures available. The intestinal microbiota provides colonization resistance to the host against invading pathogens [3–5]. One of the major mechanisms of microbiota-mediated colonization resistance is the production of a variety of metabolites that collectively restrict the proliferation of pathogens through direct inhibition of the pathogens or indirect modulation of the host response [3–5].

Butyrate is a major short-chain fatty acid (SCFA) produced by a large group of intestinal bacteria [6, 7], while deoxycholic acid (DCA) is a secondary bile acid metabolized from primary bile acids such as cholic acid by specialized bacteria [8, 9]. Once produced, butyrate and DCA exert a myriad of beneficial effects to the host. Butyrate is well known for its anti-inflammatory, barrier protective, and immunomodulatory properties [6, 7], while secondary bile acids possess antibacterial and largely anti-inflammatory activities [8, 9]. Both butyrate and bile acids are also capable of enhancing host innate immunity by inducing the synthesis of host defense peptides (HDPs) [10–15]. Additionally, butyrate and DCA have been shown to be effective in alleviating NE in chickens separately [16–18]. However, the synergy between butyrate and DCA in HDP induction and NE resistance is currently unknown.

To evaluate the potential synergistic effect of butyrate and DCA on HDP induction, we treated chicken HD11 macrophages and jejunal explants with butyrate and DCA, either individually or in combination, followed by analysis of HDP gene expression using reverse transcription-quantitative PCR (RT-qPCR). Additionally, we supplemented the diet with butyrate and DCA to assess their effectiveness in protecting chickens against NE. Our results showed that DCA positively cooperates with butyrate to enhance HDP induction and NE resistance, suggesting a synergistic effect between these two major classes of intestinal microbiota-derived metabolites on host defense. This outcome also underscores the potential of combining DCA and butyrate as an alternative to antibiotics for disease control and prevention.

## Materials and methods

### Culture and stimulation of chicken HD11 cells

Chicken HD11 macrophages were cultured in complete RPMI 1640 (HyClone, Logan, UT, USA) containing 10% fetal bovine serum (Atlanta Biologicals, Flowery Branch, GA, USA), 100 units/mL penicillin and 100 µg/mL streptomycin (Lonza, Walkersville, MD, USA), and seeded at 2 × 10^6^ cells/well in 6-well tissue culture plates (Santa Cruz Biotechnology, Dallas, TX, USA) overnight at 37 °C and 5% CO_2_. Cells were then stimulated with different concentrations of DCA (Cayman Chemical, Ann Arbor, MI, USA) and sodium butyrate (NaB) (Santa Cruz Biotechnology) in triplicate and incubated for 24 h at 37 °C and 5% CO_2_. A minimum of 2–3 independent experiments were conducted to ensure the reproducibility of the results.

### Culture and stimulation of chicken jejunal explants

Chicken jejunal explants were obtained by preparing approximately 1-cm^2^ segments of the distal jejunum from 1- to 2-week-old broiler chickens and washed with ice-cold PBS containing 100 units/mL penicillin, 100 µg/mL streptomycin, and 100 µg/mL gentamicin (HyClone), as we previously described [19–21]. Tissue explants were placed individually in 6-well tissue culture plates in RPMI 1640 containing 20 mmol/L HEPES (HyClone), 10% FBS, 100 units/mL penicillin, 100 µg/mL streptomycin, and 100 µg/mL gentamicin, followed by stimulation with different concentrations of DCA and NaB in triplicate. The tissue culture plates were then placed in a hypoxia chamber (Catalog # 27310, StemCell Technologies, Vancouver, BC, Canada) and flushed for 2 min with 95% O_2_ and 5% CO_2_. Cells were incubated at 37 °C for 24 h. At least two independent experiments were conducted to ensure the reproducibility of the results.

### RT-qPCR analysis of gene expression

Following incubation with DCA and NaB, chicken HD11 cells and jejunal explants were lysed in RNAzol RT (Molecular Research Center, Cincinnati, OH, USA) for extraction of total RNA. RNA concentrations were quantified using Nanodrop One Spectrophotometer (Thermo Fisher Scientific, Waltham, MA, USA), and 0.3-µg RNA was used for reverse transcription using iScript cDNA Synthesis kit (Bio-Rad, Hercules, CA, USA) according to the manufacturer’s instructions. Quantitative PCR was performed with iTaq Universal SYBR Green Supermix (Bio-Rad) and gene-specific primers as we described [22–24]. Primer sequences were described previously [11, 19, 22], and forward and reverse primers were added to a final concentration of 500 nmol/L. The qPCR reactions were performed in CFX96 Real-time PCR Detection System (Bio-Rad) with initial 95 °C for 30 s and 40 cycles of 94 °C for 5 s and 60 °C for 30 s, followed by melt curve analysis. Gene expression levels were calculated with the 2^−ΔΔCt^ method using glyceraldehyde 3-phosephate dehydrogenase (*GAPDH*) as the reference gene.

### Chicken NE trials

Three NE trials were conducted to investigate the efficacy of DCA and/or NaB in protecting chickens against NE using a co-infection model of *Eimeria maxima* and *Clostridium perfringens* as we previously described [19–21]. All animal procedures were approved by the Institutional Animal Care and Use Committee of Oklahoma State University under protocol number IACUC-21-65. In the first trial, a total of 150 day-of-hatch male Cobb chicks were obtained from Cobb-Vantress Hatchery (Siloam Springs, AR, USA) and housed in an environmentally controlled room under standard management as recommended by Cobb-Vantress. Chickens were weighed and randomly assigned to 10 birds/pen and 3 pens per treatment. Animals had ad libitum access to a standard corn-soybean meal mash starter diet (21% crude protein) supplemented with or without three different concentrations (0.5, 1.0, and 1.5 g/kg) of DCA (Cayman Chemical, Ann Arbor, MI, USA) throughout the entire trial. On d 10, all but 3 pens of chickens were orally challenged with 5 × 10^3^ sporulated *E. maxima* oocysts (kindly provided by John R. Barta, University of Guelph, Canada) in 1 mL saline after overnight fasting. To encourage oocyst recycling, water was sprayed onto wood shavings twice a day on d 10 and 11. On d 14, approximately 4–5 × 10^8^ CFU of *C. perfringens* strain Brenda B, carrying *netB* and *tpeL* toxin genes (kindly provided by Lisa Bielke at Ohio State University, USA) was orally inoculated in 2 mL fluid thioglycollate (FTG) broth (Thermo Fisher Scientific) after overnight fasting. Mock-infected control chickens in 3 pens were fed basal diet and inoculated with 1 mL saline and 2 mL FTG broth on d 10 and 14, respectively. All birds were weighed individually on d 10 and 17, and the survival was monitored twice daily till the end of the trial on d 17. Chickens that were unable to stand, move, eat, or drink were euthanized by CO_2_ asphyxiation to minimize undue pain. All surviving animals were sacrificed on d 17 and the jejunum was scored for gross lesions of NE using a 6-point scoring system as described [25].

In the second NE trial, a total of 210 day-of-hatch male Cobb chicks were obtained and fed the mash starter diet with or without supplementation with 1 g/kg microencapsulated sodium butyrate (NaB; King Technica, Hangzhou, China), two concentrations of DCA (0.75 or 1.5 g/kg), or a combination of 1 g/kg microencapsulated NaB with either concentration of DCA. Each group consisted of three floor pens with 10 chickens/pen. Six groups of chickens were sequentially infected with 5 × 10^3^ *E. maxima* in 1 mL saline on d 10 and 4–5 × 10^8^ CFU of *C. perfringens* in 2 mL FTG, respectively, as described in the first trial, while the seventh group was mock-infected with 1 mL saline and 2 mL FTG on d 10 and 14, respectively. Animals were weighed individually on d 10 and 17, and the survival was monitored twice daily till the end of the trial on d 17. All surviving animals were sacrificed on d 17 and the jejunal segment was scored for gross lesions of NE using a 6-point scoring system [25].

A third NE trial was conducted the same as the second trial to confirm the protection of chickens by DCA in combination with NaB. On d 17, all surviving animals were sacrificed, and approximately 0.5 mg of the digesta from the proximal ileum and 0.3 mg of the cecal digesta were aseptically collected in two separate tubes randomly from 12 animals/treatment and 4 animals/pen and snap-frozen in liquid nitrogen. The jejunal segment of the intestine was also scored for gross lesions of NE using a 6-point scoring system [25].

### Gut microbiome analysis

Microbial genomic DNA was extracted from the ileal and cecal digesta using ZR Fecal DNA MicroPrep and MiniPrep kit (Zymo Research, Irvine, CA, USA), respectively. The resulting DNA concentration and purity were assessed using Nanodrop One Spectrophotometer (Thermo Fisher Scientific). High quality DNA was shipped on dry ice to Novogene (Beijing, China) for PE250 deep sequencing of the V3–V4 region of bacterial 16S rRNA gene using primers (341F: 5´-CCTAYGGGRBGCASCAG-3´ and 806R: 5´-GGACTACNNGGGTATCTAAT-3´) on an Illumina platform. PCR amplification and library preparation were performed by Novogene (Beijing, China) using NEBNext^®^ Ultra™ Library Prep kit (New England Biolabs, Ipswich, MA, USA).

Downstream bioinformatic analysis was conducted as we previously described [20, 26, 27]. Briefly, raw sequencing reads were analyzed in QIIME 2 v2020.11 [28]. Adaptor, barcode, and primer sequences were removed before downstream analysis using the “Cut-Adapt” plug-in in QIIME2 [29]. Forward and reverse reads of each sample were then joined, and quality control analysis was performed. Deblur algorithm v2022.8.0 [30] was used to denoise by removing low quality reads to produce filtered amplicon sequence variants (ASVs). ASVs were then classified using the Ribosomal Database Project 16S rRNA training set v18 and Bayesian classifier [31]. The taxons with bootstrap confidence of < 80% were assigned the name of the last confidently assigned taxonomic level followed by “_undefined”. ASVs appearing in < 5% of samples were removed from further downstream analysis. The top 50 ASVs and all differentially enriched bacteria were further confirmed and reclassified, based on a recent EzBioCloud 16S database v2021.07.07 [32].

Analysis and visualization of α- and β-diversities of the microbiota composition were conducted in R v3.6.3 [33], utilizing the R ‘phyloseq’ package v1.30.0 [34]. To visualize the overall biodiversity and complexity within ileal samples, the number of ASVs, Pielou’s evenness index, and Shannon index were used to calculate and display the overall α-diversity, richness, and evenness. The β-diversity was determined using weighted and unweighted UniFrac distances [35]. Differential enrichment of bacteria between different groups was determined using linear discriminant analysis (LDA) effect size (LEfSe), with the all-against-all multiclass analysis using *P* < 0.05 and a logarithmic LDA score of ≥ 3.0 as the threshold as described [36].

### Statistical analysis

Data analysis and graphical visualization were implemented in GraphPad Prism 9 (GraphPad, La Jolla, CA, USA) or RStudio v1.2.1578 (RStudio, Boston, MA, USA). Statistical significance was measured using parametric or non-parametric methods, depending on the normality of the data determined by the Shapiro–Wilk test. Results were presented as mean ± standard error of the mean (SEM). The gene expression fold changes and chicken body weight gain were compared among treatments using one-way ANOVA and post hoc Tukey’s test, while lesion scores, bacterial α-diversity and relative abundance were compared using the Kruskal–Wallis test and post hoc Wilcoxon rank-sum test. Animal survival rates were compared using the log-rank test. Bacterial β-diversity was compared among groups by permutational multivariate analysis of variance (PERMANOVA) with 999 permutations using ‘vegan’ package v2.5.6 available in R. *P* < 0.05 was considered statistically significant.

## Data deposition

Raw sequencing reads of this study was deposited in the NCBI GenBank SRA database under BioProject ID: PRJNA1018846.

## Results

### Regulation of HDP and tight junction protein gene expression in HD11 cells and jejunal explants by DCA and NaB

**Fig. 1:**
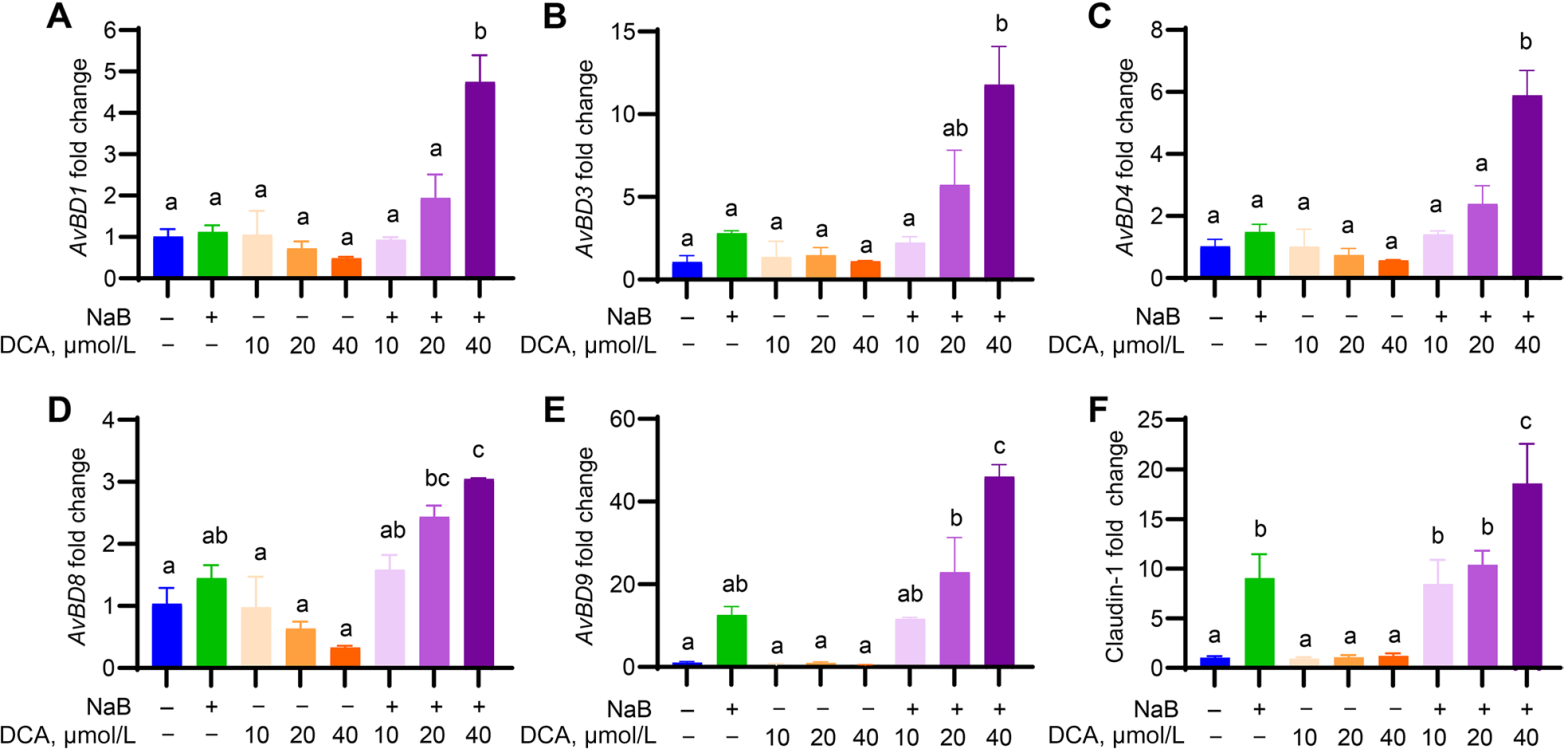
Synergy between DCA and butyrate in inducing avian β-defensin (*AvBD*) and claudin-1 gene expression in chicken macrophages. Chicken HD11 macrophages were stimulated in triplicate with 1 mmol/L sodium butyrate (NaB) in the presence or absence of indicated concentrations (µmol/L) of deoxycholic acid (DCA) for 24 h, followed by reverse transcription-quantitative PCR analysis (RT-qPCR) of mRNA expression of *AvBD1* (**A**), *AvBD3* (**B**), *AvDB4* (**C**), *AvBD8* (**D**), *AvBD9* (**E**), and claudin-1 (**F**). Data shown are mean ± SEM of a representative of 2–3 independent experiments. Means not sharing a common superscript letter denote statistical significance (*P* < 0.05) based on one-way ANOVA and post hoc Tukey’s test

To investigate a possible synergy between DCA and butyrate in modulating major HDP gene expression, chicken HD11 cells were treated with DCA and NaB individually or in combination, followed by RT-qPCR analysis of HDP mRNA expression levels. DCA alone showed no obvious induction of chicken HDP genes at the concentrations tested, while 1 mmol/L NaB was relatively potent (Fig. 1A–E). DCA, when combined with NaB, significantly enhanced the magnitude of HDP induction over either compound alone (Fig. 1A–E). Desirably, DCA also synergized with NaB in inducing the expression of claudin-1 (Fig. 1F), a major gene involved in the assembly of tight junction [37]. It is noteworthy that the synergy between DCA and NaB was not uniformly obvious for all chicken HDP and barrier function genes. For example, avian β-defensin 6 (*AvBD6*), *AvBD7*, *AvBD10*, tight junction protein-1, and mucin-2 genes were not synergistically induced (data not shown).

**Fig. 2:**
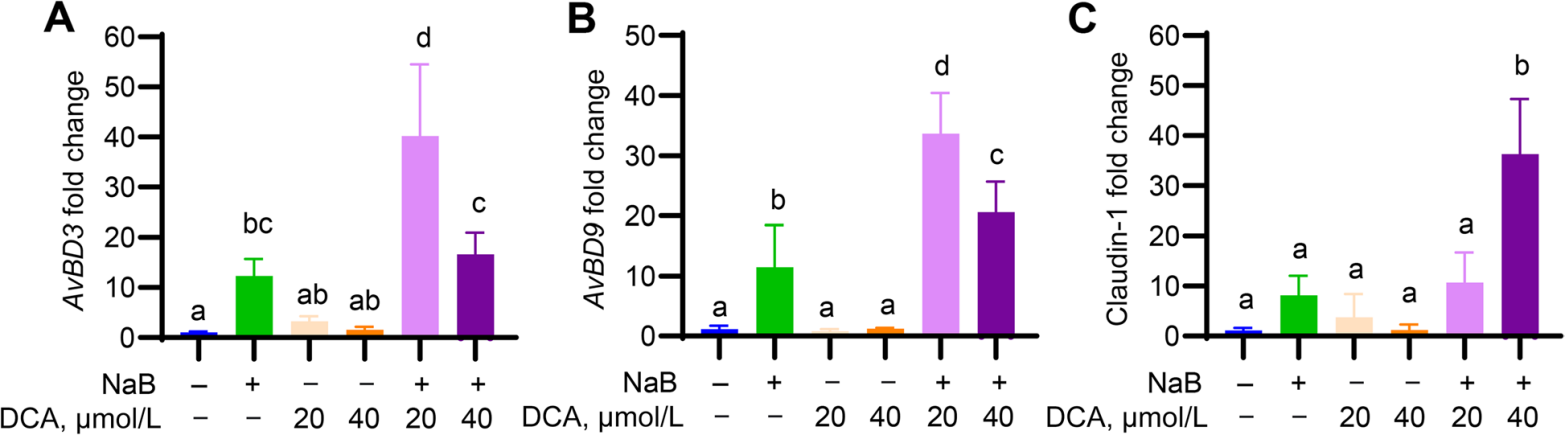
Synergy between DCA and butyrate in inducing avian β-defensin (*AvBD*) and claudin-1 gene expression in chicken jejunal explants. Chicken jejunal explants were stimulated in triplicate with 4 mmol/L NaB with or without indicated concentrations (µmol/L) of DCA for 24 h, followed by RT-qPCR analysis of mRNA expression of *AvBD3* (**A**), *AvBD9* (**B**), and claudin-1 (**C**). Data shown are mean ± SEM of 2–3 independent experiments. Means not sharing a common superscript letter denote statistical significance (*P* < 0.05) based on one-way ANOVA and post hoc Tukey’s test

To further confirm the DCA/NaB synergy, chicken jejunal explants were prepared and stimulated with DCA and NaB separately or in combination. As in HD11 cells, DCA alone showed a minimum impact on HDP gene expression, but a combination of 4 mmol/L NaB and 20 μmol/L DCA gave a significant increase the gene expression of both *AvBD3* (Fig. 2A) and *AvBD9* (Fig. 2B). Similarly, claudin-1 mRNA expression was also synergistically induced by 4 mmol/L NaB and 40 μmol/L DCA (Fig. 2C). Collectively, these results in two different cell types suggested a synergy between DCA and NaB in the upregulation of several, but not all, HDP genes. Both compounds also had a positive impact on the gene expression of certain tight junction proteins, implying their potential positive impact on mucosal barrier function.

### Alleviation of NE in chickens by DCA and NaB

DCA has been shown to alleviate NE [16–18]. To confirm the role of DCA in NE mitigation, 0.5, 1.0, or 1.5 g/kg DCA was supplemented to the basal diet on the day-of-hatch, prior to NE induction. Clearly, a concentration-dependent protection of chickens from NE was observed. While only 35% chickens survived without dietary intervention, 100% chickens were protected by 1.5 g/kg DCA (Fig. S1A). Furthermore, DCA caused a concentration-dependent reversal of growth retardation (Fig. S1B). Consistently, the intestinal lesion severity was also reduced by DCA in a concentration-dependent manner, with the highest reduction seen with 1.5 g/kg DCA (Fig. S1C).

To evaluate a possible synergy between DCA and NaB in alleviating NE, two different concentrations of DCA (0.75 and 1.5 g/kg) and 1 g/kg microencapsulated NaB were supplemented to the basal diet individually or in combination throughout the trial. Again, 1.5 g/kg DCA provided the best protection against NE, achieving a survival rate of 98% as compared to 80% survival with no intervention (Fig. S2A). While NaB alone had a negligible effect, 1.5 g/kg DCA was superior to 0.75 g/kg DCA (Fig. S2A). However, 0.75 g/kg DCA in combination with NaB improved the survival rate to the level of 1.5 g/kg DCA (Fig. S2A), implying a synergistic effect. Consistently, intestinal lesions of the 0.75 g/kg DCA/NaB group were the least severe among all groups, with only 15% birds showing severe intestinal lesions, while 44% birds in the NE group had extensive score-6 lesions (Fig. S2B). It is noted that a higher concentration (1.5 g/kg) of DCA combined with NaB gave slightly reduced survival and more severe intestinal lesions than the 0.75 g/kg DCA/NaB group.

**Fig. 3:**
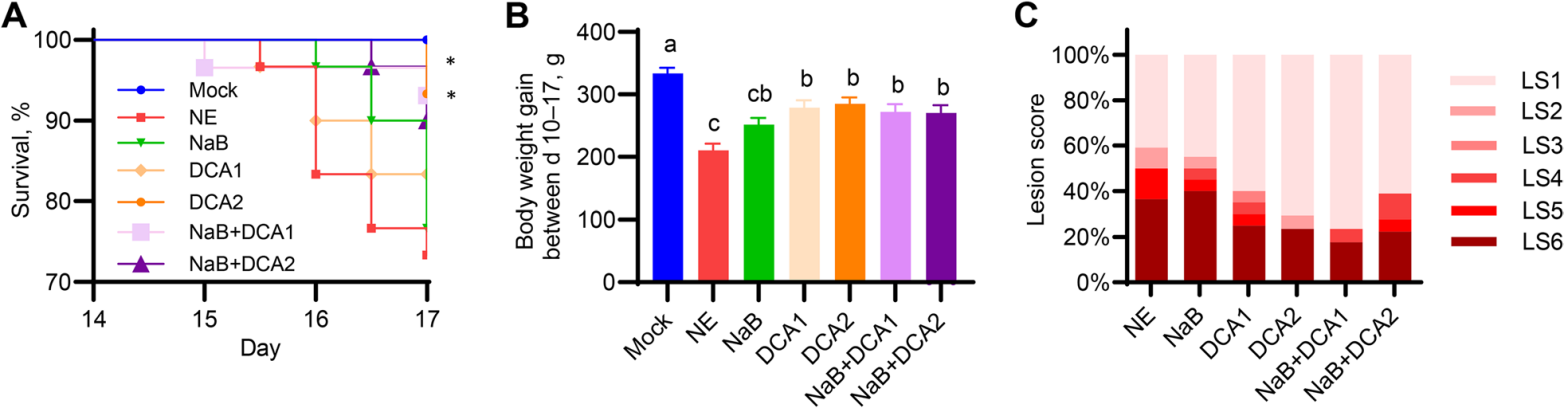
Synergy between DCA and butyrate in alleviating necrotic enteritis (NE) in broiler chickens. A total of 210 day-of-hatch male Cobb broilers were allotted to one of seven groups (*n* = 30) supplemented with or without 1 g/kg NaB, 0.75 g/kg DCA (DCA1), 1.5 g/kg DCA (DCA2), or a mixture of 1 g/kg NaB and 0.75 g/kg DCA (NaB + DCA1) or 1 g/kg NaB and 1.5 g/kg DCA (NaB + DCA2). Six groups of animals were subjected to NE by sequential infections with *Eimeria maxima* on d 10 and *Clostridium perfringens* on d 14, while the remaining one group of 30 chickens were mock-infected as negative controls. **A** Animal survival (%) between d 14–17. Note that two groups (DCA2 and NaB + DCA1) are significantly different from the NE group (^*^P < 0.05) based on the log-rank test. **B** Individual body weight gains of surviving animals between d 10–17. Data shown are mean ± SEM. Means not sharing a common superscript letter denote statistical significance (*P* < 0.05) based on one-way ANOVA and post hoc Tukey’s test. **C** Frequency (%) of jejunal lesion scores on d 17

To ensure the reproducibility of the results, a third trial was conducted in the same manner as the second trial. Consistently, 1.5 g/kg DCA provided significant protection and was better than 0.75 g/kg DCA (Fig. 3A). Furthermore, a combination of 0.75 g/kg DCA and NaB gave an identical survival rate to 1.5 g/kg DCA. On the other hand, 1.5 g/kg DCA combined with NaB showed slightly reduced survival (Fig. 3A). The three most effective treatments with the lowest lethality were also the best in reversing NE-induced growth retardation (Fig. 3B). Similar to the second trial, the 0.75 g/kg DCA/NaB group showed the least severe intestinal lesions, while 1.5 g/kg DCA with or without butyrate also gave relatively mild lesion scores (Fig. 3C), confirming the consistency of the DCA/NaB synergy in live animals.

### Impact of DCA and NaB on the cecal microbiome

To understand the influence of supplementing DCA and NaB on the gut microbiome in the context of NE, the V3–V4 region of the bacterial 16S rRNA gene in the cecal and ileal digesta of all surviving animals in the third trial was deep-sequenced and analyzed. Following quality control, 15,536,083 high-quality sequencing reads were obtained, with an average of 105,688 ± 2,499 sequences per sample. After removing ASVs present in < 5% of samples, 501 and 563 ASVs were obtained in the cecal and ileal microbiota samples, respectively.

**Fig. 4:**
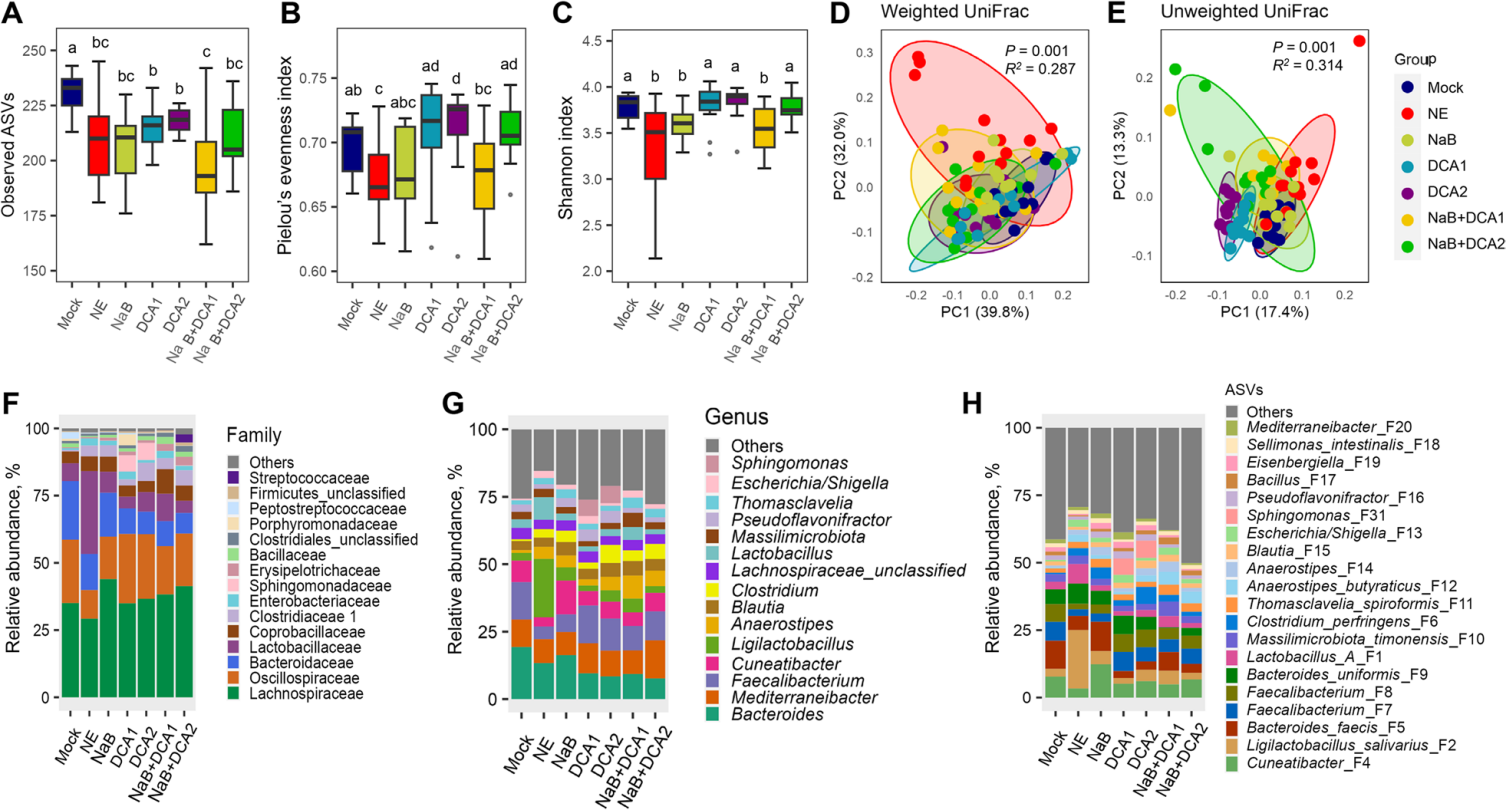
The diversity and composition of the cecal microbiota in healthy and NE-infected chickens supplemented with DCA or butyrate. Day-of-hatch male Cobb broilers were supplemented with or without 1 g/kg NaB, 0.75 g/kg DCA (DCA1), 1.5 g/kg DCA (DCA2), or a mixture of 1 g/kg NaB and 0.75 g/kg DCA (NaB + DCA1) or 1 g/kg NaB and 1.5 g/kg DCA (NaB + DCA2). Six groups of animals were subjected to NE, while the remaining group was mock-infected. The cecal digesta were randomly collected from 12 surviving animals/group on d 17 and subjected to 16S rRNA gene sequencing. Observed ASVs (**A**), Pielou’s evenness index (**B**), and Shannon index (**C**) were estimated and visualized using box and whisker plots. Significance was measured using the Kruskal–Wallis test and post hoc pairwise Wilcoxon rank-sum test. Different superscripts denote significance (*P* < 0.05) in pairwise comparisons. Principal coordinates analysis (PCoA) plots of weighted (**D**) and unweighted UniFrac distances (**E**). Significance was determined using PERMANOVA. Relative abundances of the top 15 families (**F**), top 15 genera (**G**), and top 20 ASVs (**H**) in the cecal microbiota are shown

In the cecum, NE infection caused a significant decrease in the richness, evenness, and overall α-diversity (Fig. 4A–C). Although all dietary treatments failed to restore the richness, most were largely capable of restoring the evenness and overall diversity of the cecal microbiota (Fig. 4A–C). NE also caused a significant shift of β-diversity of the cecal microbiota in both weighted and unweighted UniFrac distances, and none of the treatments were able to completely restore the cecal microbiota back to normal (Fig. 4D and E). It is also evident that the cecal microbiotas of various dietary treatments were mostly different from each other (Table S1). Compositionally, Lachnospiraceae*,* Oscillospiraceae*,* Lactobacillaceae, and Bacteroidaceae accounted for approximately 90% of total bacteria in the cecum (Fig. 4F), which were represented by a large number of bacterial genera with no particular dominance (Fig. 4G). Strikingly, *Ligilactobacillus salivarius* (F2) was below 3% in the cecum of mock-infected chickens and surged to 22% in NE-infected chickens, but was largely restored in response to various dietary treatments (Fig. 4H).

**Fig. 5:**
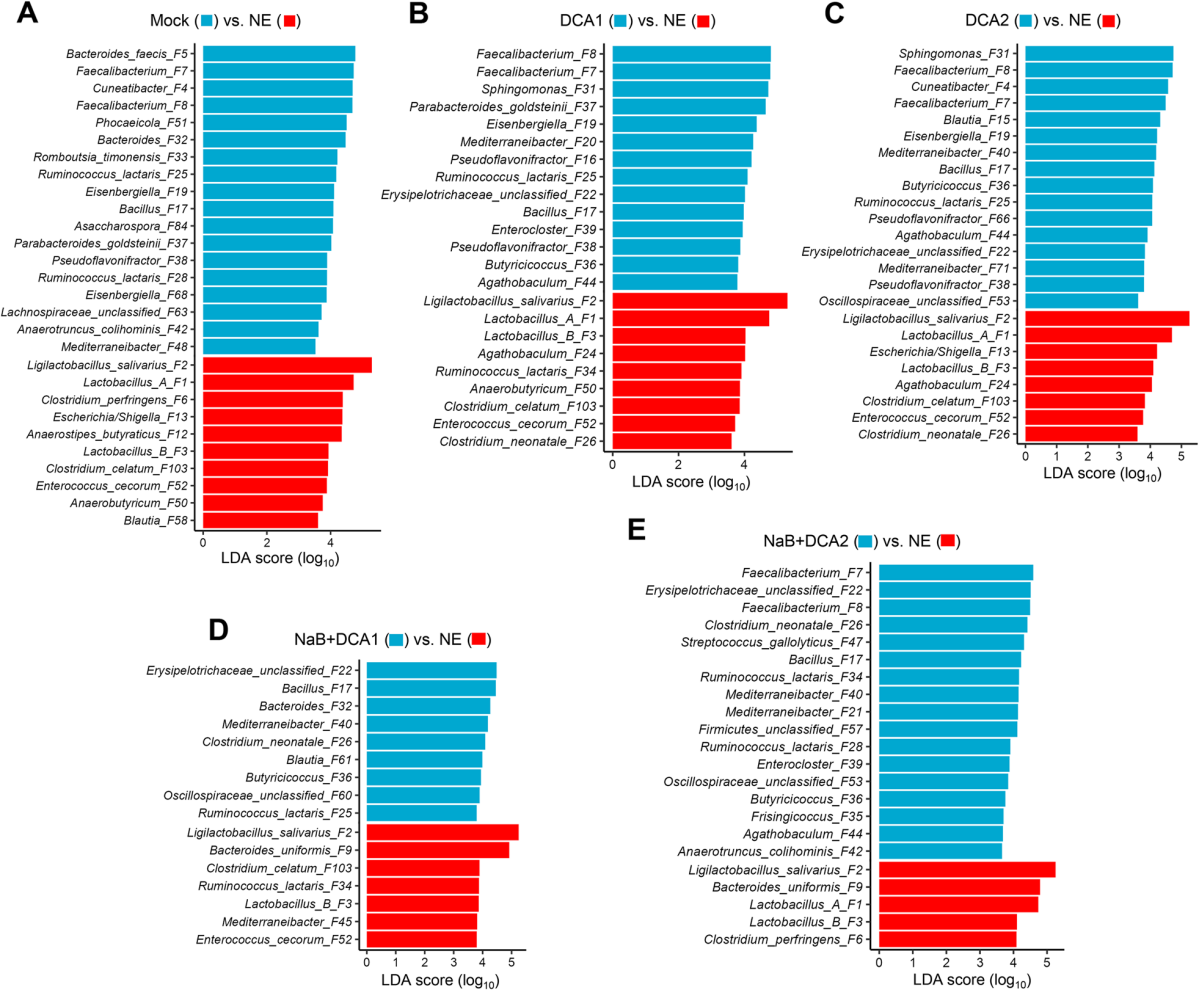
Differential enrichment of the cecal microbiota in healthy and NE-infected chickens supplemented with DCA or butyrate. Day-of-hatch male Cobb broilers were supplemented with or without 1 g/kg NaB, 0.75 g/kg DCA (DCA1), 1.5 g/kg DCA (DCA2), or a mixture of 1 g/kg NaB and 0.75 g/kg DCA (NaB + DCA1) or 1 g/kg NaB and 1.5 g/kg DCA (NaB + DCA2). Six groups of animals were subjected to NE, while the remaining group was mock-infected. The cecal digesta were collected from 12 animals/group on d 17. LEfSe analysis was performed with the top 50 ASVs in the d 17 cecal digesta among different groups of chickens (*n* = 12), with cut-offs of *P* < 0.05 and a logarithmic LDA score of 3.0

To identify differential enrichment of the cecal microbiota among different treatments, LEfSe analysis [36] was performed with the 50 most abundant bacterial ASVs. A characteristic increase of *C. perfringens* (F6), *Escherichia/Shigella* (F13), and *Enterococcus cecorum* (F52) was observed in response to NE, along with a significant diminishment of a large number of SCFA-producing bacteria such as *Bacteroides faecis* (F5), *Faecalibacterium* (F7 and F8), and *Cuneatibacter* (F4) (Fig. 5A). In contrast, Group A *Lactobacillus* (F1), Group B *Lactobacillus* (F3), and *L. salivarius* (F2) were significantly enriched in the NE-infected cecum (Fig. 5A). It is noted that several less abundant SCFA-producing bacteria such as *Anaerostipes butyraticus* (F12), *Anaerobutyricum* (F50), *Blautia* (F58), and *Clostridium celatum* (F103) were promiscuously enriched in response to NE (Fig. 5A). Among NE-infected chickens, major SCFA-producing bacteria such as *Faecalibacterium* (F7 and F8) were enriched, while three major lactobacilli (F1, F2, and F3) were reduced in response to both concentrations of DCA (Fig. 5B and C). Interestingly, *Sphingomonas* (F31) was obviously more abundant in both DCA groups (Fig. 5B and C). Three major lactobacilli (F2, F3, and/or F1) were similarly reduced in response to butyrate in combination with either concentration of DCA (Fig. 5D and E).

**Fig. 6:**
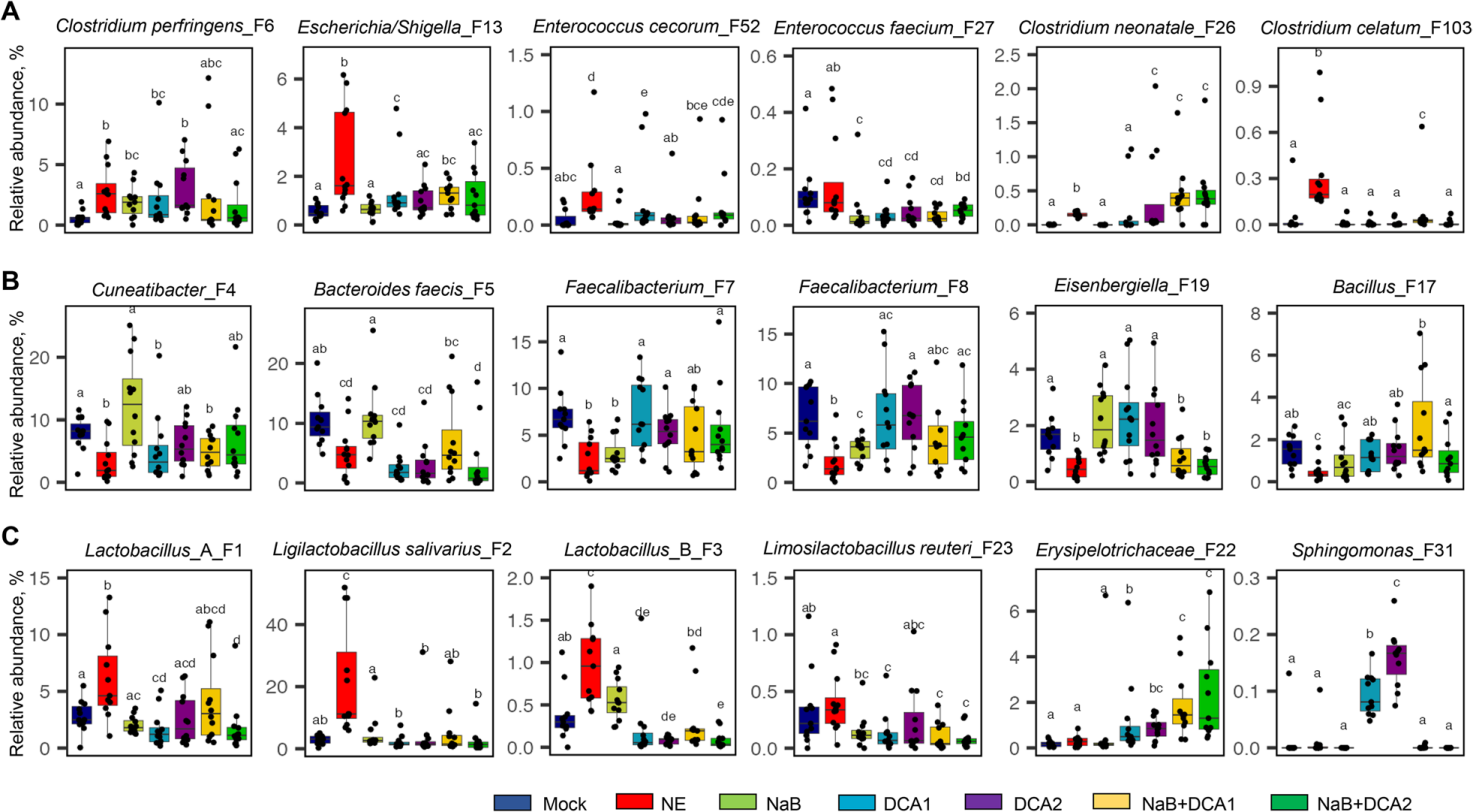
Relative abundances of representative cecal bacteria showing significant enrichment in healthy and NE-infected chickens supplemented with DCA or butyrate. Day-of-hatch male Cobb broilers were supplemented with or without 1 g/kg NaB, 0.75 g/kg DCA (DCA1), 1.5 g/kg DCA (DCA2), or a mixture of 1 g/kg NaB and 0.75 g/kg DCA (NaB + DCA1) or 1 g/kg NaB and 1.5 g/kg DCA (NaB + DCA2). Six groups of animals were subjected to NE, while the remaining group was mock-infected. The cecal digesta were collected from 12 animals/group on d 17 and subjected to 16S rRNA gene sequencing. **A** Relative abundances (%) of *C. perfringens*, pathobionts, and two *Clostridium* species. **B** Relative abundances (%) of SCFA-producing bacteria and a *Bacillus* species. **C** Relative abundances (%) of lactobacilli, an *Erysipelotrichaceae* species, and a *Sphingomonas* species. In the box and whisker plots, each box indicates median, 25^th^ and 75^th^ percentiles, while whiskers extend to 1.5 interquartile range. Significance was measured using the Kruskal–Wallis test and post hoc pairwise Wilcoxon rank sum test, and the significance (*P* < 0.05) was denoted by different superscripts

Overall, *C. perfringens* (F6), *Escherichia/Shigella* (F13), and *E. cecorum* (F52) were significantly increased in response to NE, while all dietary treatments appeared to largely restore them to healthy levels (Fig. 6A). Although it was comparable between mock- and NE-infected chickens, *E. faecium* (F27) was further suppressed by dietary treatments (Fig. 6A). Interestingly, two related *Clostridium* species (F26 and F103) responded differently to dietary interventions. While both were significantly enriched by NE, *C. neonatale* (F26) was further elevated by three most effective dietary interventions (1.5 g/kg DCA and two combination groups), and *C. celatum* (F103) was largely returned to normal levels (Fig. 6A).

Additionally, several major SCFA-producing bacteria such as *Cuneatibacter* (F4), *B. faecis* (F5), *Faecalibacterium* (F7 and F8), and *Eisenbergiella* (F19) were significantly suppressed by NE, while effective dietary treatments tended to restore *Cuneatibacter* (F4) and *Faecalibacterium* (F7 and F8), but not *B. faecis* (F5) (Fig. 6B). A *Bacillus* species (F17) was almost abolished by NE, but largely restored by all dietary treatments (Fig. 6B). To our surprise, all major species of lactobacilli in the cecum (F1, F2, and F3) were significantly increased in response to NE and largely restored or even further suppressed by effective dietary treatments (Fig. 6C). A less abundant *L. reuteri* (F23) also appeared to be reduced below normal levels by dietary compounds, although NE has no significant influence on its abundance. Interestingly, a member of *Erysipelotrichaceae* (F22) was minimally present in both mock- and NE-infected chickens, but appeared to be dose-dependently increased in response to DCA and its combination with butyrate (Fig. 6C). On the other hand, a *Sphingomonas* species (F31) was minimal in healthy animals, but dose-dependently enriched in both DCA groups, but no other groups (Fig. 6C).

### Impact of DCA and NaB on the ileal microbiome

**Fig. 7:**
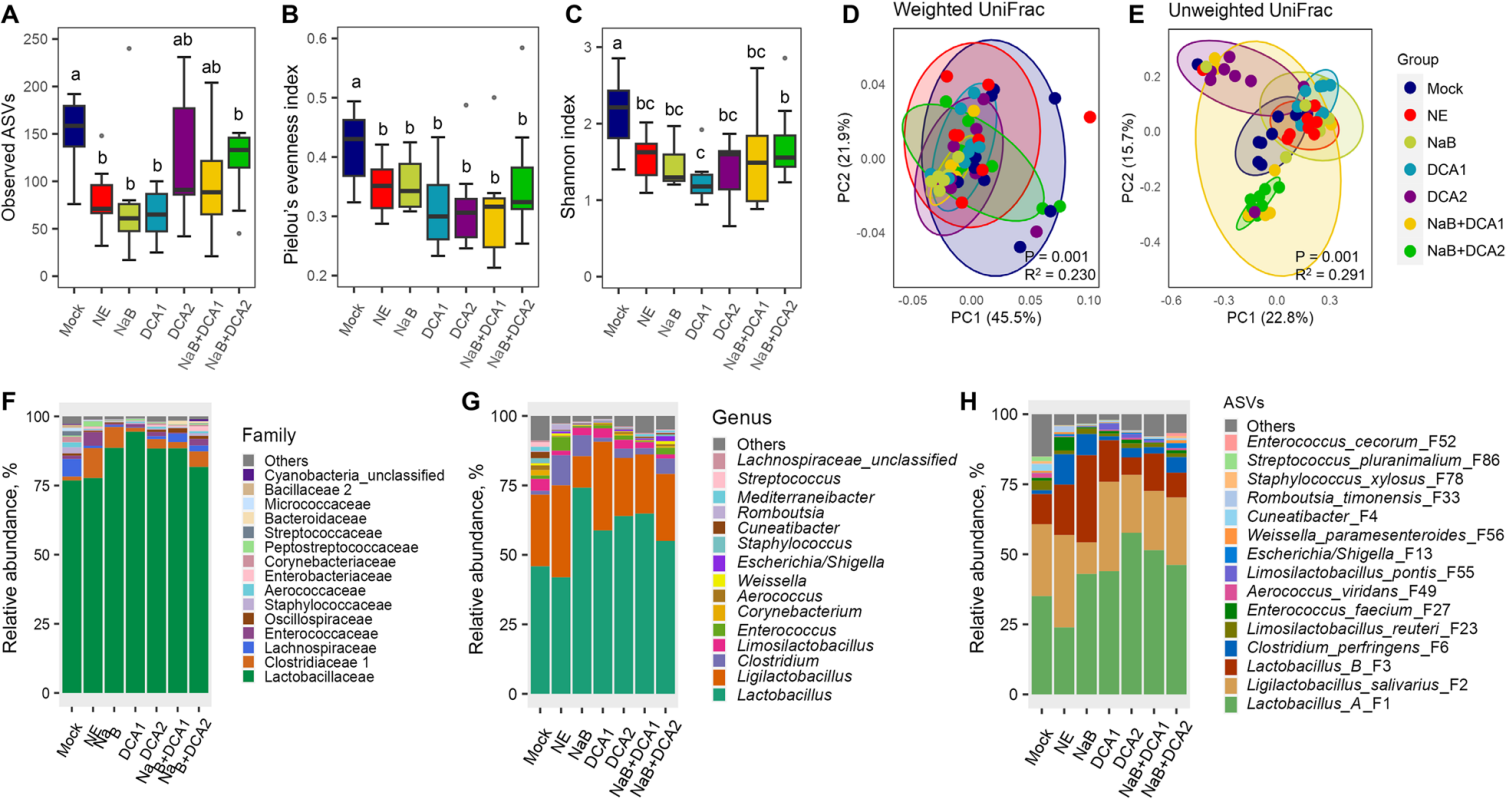
The diversity and composition of the ileal microbiota in healthy and NE-infected chickens supplemented with DCA or butyrate. Day-of-hatch male Cobb broilers were supplemented with or without 1 g/kg NaB, 0.75 g/kg DCA (DCA1), 1.5 g/kg DCA (DCA2), or a mixture of 1 g/kg NaB and 0.75 g/kg DCA (NaB + DCA1) or 1 g/kg NaB and 1.5 g/kg DCA (NaB + DCA2). Six groups of animals were subjected to NE, while the remaining group was mock-infected. The proximal ileal digesta were randomly collected from 12 surviving animals/group on d 17 and subjected to 16S rRNA gene sequencing. Observed ASVs (**A**), Pielou’s evenness index (**B**), and Shannon index (**C**) were estimated and visualized using box and whisker plots. Significance was measured using the Kruskal–Wallis test and post hoc pairwise Wilcoxon rank-sum test. Different superscripts denote significance (*P* < 0.05) in pairwise comparisons. Principal coordinates analysis (PCoA) plots of weighted (**D**) and unweighted UniFrac distances (**E**). Significance was determined using PERMANOVA. Relative abundances of the top 15 families (**F**), top 15 genera (**G**), and top 20 ASVs (**H**) in the ileal microbiota are shown

NE infection significantly reduced bacterial richness in the ileum as indicated by observed ASVs, but the reduction was largely reversed by three most effective dietary interventions (1.5 g/kg DCA and two combination groups) (Fig. 7A). On the other hand, non-effective treatments such as NaB alone or 0.75 g/kg DCA showed little effect in reversing bacterial richness. The evenness and Shannon index of the ileal microbiota were also significantly reduced by NE, but none of the treatments were capable of rescuing them (Fig. 7B and C). NE also triggered a significant shift in β-diversity as indicated by weighted and unweighted UniFrac distances (Fig. 7D and E, and Table S2). Compositionally, Lactobacillaceae was the most abundant family in the ileum, accounting for 72% of the total bacterial population in healthy chickens (Fig. 7F). In fact, Lactobacillaceae primarily consisted of four ASVs, namely Group A *Lactobacillus* (F1), *L. salivarius* (F2), Group B *Lactobacillus* (F3), and *Limosilactobacillus reuteri* (F23), representing 32%, 27%, 10%, and 3% of all ileal bacteria in healthy chickens, respectively (Fig. 7G and H). Unsurprisingly, *C. perfringens* (F6) became obviously more abundant in NE and several other dietary treatment groups, relative to mock-infected animals (Fig. 7H).

**Fig. 8:**
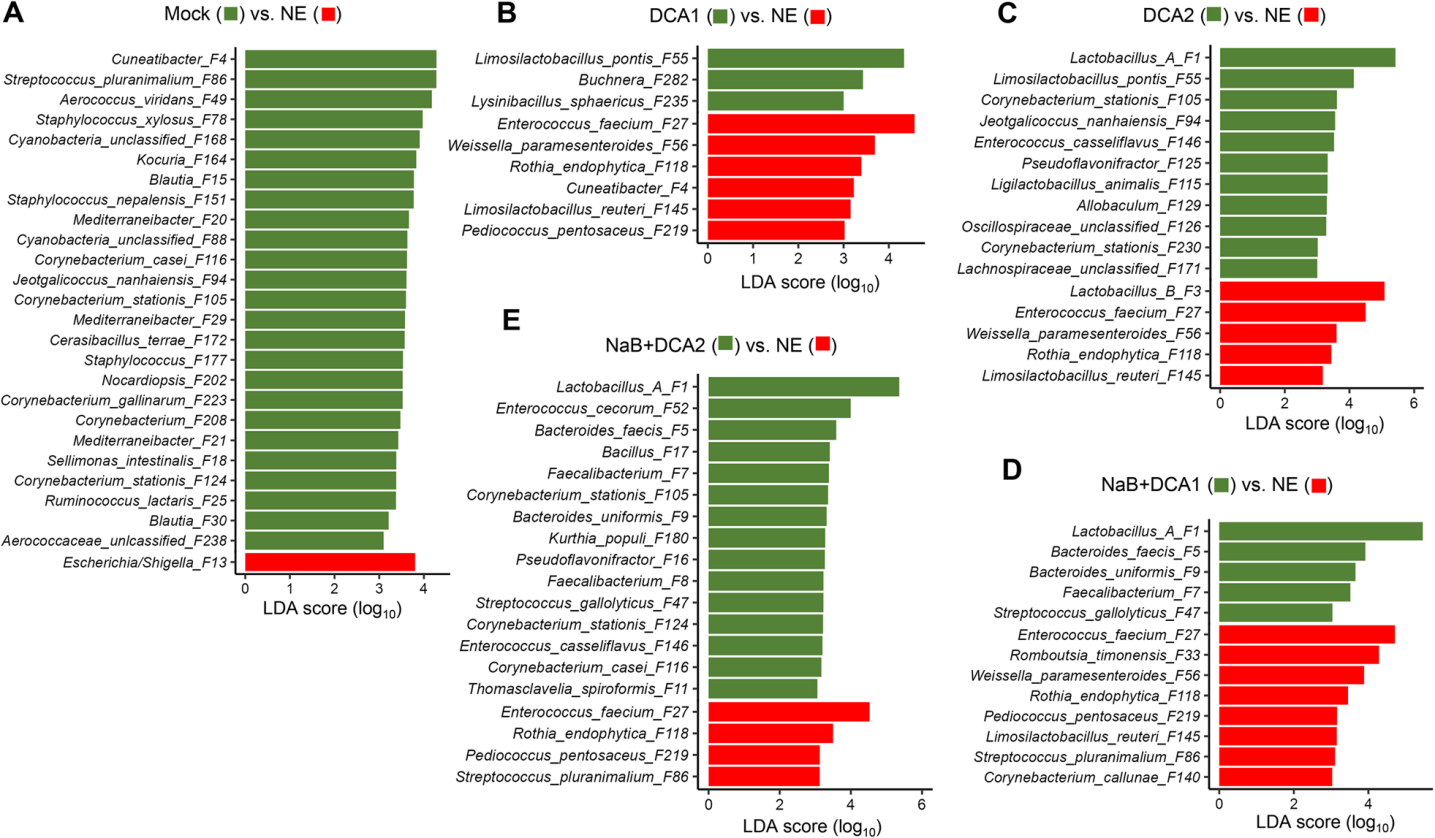
Differential enrichment of the ileal microbiota in healthy and NE-infected chickens supplemented with DCA or butyrate. Day-of-hatch male Cobb broilers were supplemented with or without 1 g/kg NaB, 0.75 g/kg DCA (DCA1), 1.5 g/kg DCA (DCA2), or a mixture of 1 g/kg NaB and 0.75 g/kg DCA (NaB + DCA1) or 1 g/kg NaB and 1.5 g/kg DCA (NaB + DCA2). Six groups of animals were subjected to NE, while the remaining group was mock-infected. The proximal ileal digesta were randomly collected from 12 surviving animals/group on d 17 and subjected to 16S rRNA gene sequencing. LEfSe analysis was performed with the top 50 ASVs among different groups of chickens, with cut-offs of *P* < 0.05 and a logarithmic LDA score of 3.0

LEfSe analysis of the 50 most abundant bacterial ASVs in the ileum revealed an enrichment of *Escherichia/Shigella* (F13) by NE, with a significant reduction in a number of bacteria such as *Cuneatibacter* (F4) and *Streptococcus pluranimalium* (F86) (Fig. 8A), which is consistent with our earlier observation [20]. Supplementation of 0.75 and 1.5 g/kg DCA commonly enriched *Limosilactobacillus pontis* (F55) and reduced *E. faecium* (F27), *Weissella paramesenteroides* (F56), *Rothia endophytica* (F118), and *L. reuteri* (F145) in NE-infected chickens (Fig. 8B and C). Butyrate in combination with either concentration of DCA caused a significant increase of *Lactobacillus* Group A (F1), *Bacteroides faecis* (F5), *Faecalibacterium* (F7), *Bacteroides uniformis* (F9), and *Streptococcus gallolyticus* (F47), with a concurrent reduction of *E. faecium* (F27) and *R. endophytica* (F118) in the ileum of NE chickens (Fig. 8D and E). A common feature among three most effective treatment groups was a significant increase in *Lactobacillus* Group A (F1) and diminishment of *E. faecium* (F27) and *R. endophytica* (F118) (Fig. 8C–E). In comparison with two DCA groups, both butyrate/DCA combination groups showed a unique enrichment of two species of *Bacteroides* (F5 and F9), *Faecalibacterium* (F7), and *S. gallolyticus* (F47).

**Fig. 9:**
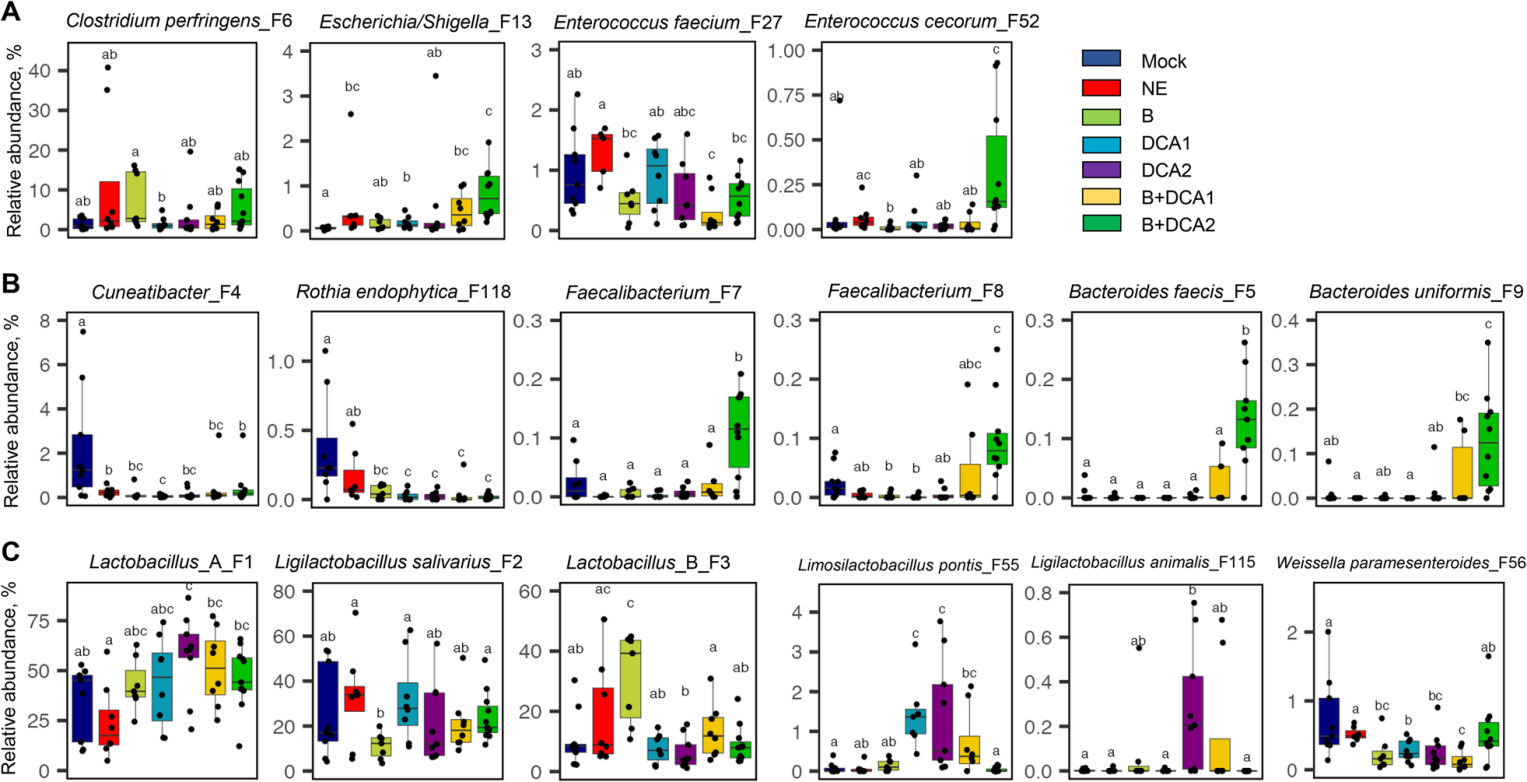
Relative abundances of representative ileal bacteria showing significant differential enrichment in healthy and NE-infected chickens supplemented with DCA or butyrate. Day-of-hatch male Cobb broilers were supplemented with or without 1 g/kg NaB, 0.75 g/kg DCA (DCA1), 1.5 g/kg DCA (DCA2), or a mixture of 1 g/kg NaB and 0.75 g/kg DCA (NaB + DCA1) or 1 g/kg NaB and 1.5 g/kg DCA (NaB + DCA2). Six groups of animals were subjected to NE, while the remaining group was mock-infected. The proximal ileal digesta were randomly collected from 12 surviving animals/group on d 17 and subjected to 16S rRNA gene sequencing. **A** Relative abundances (%) of *C. perfringens* and pathobionts. **B** Relative abundances (%) of SCFA-producing bacteria. **C** Relative abundances (%) of several lactobacilli. In the box and whisker plots, each box indicates median, 25^th^ and 75^th^ percentiles, while whiskers extend to 1.5 interquartile range. Significance was measured using the Kruskal–Wallis test and post hoc pairwise Wilcoxon rank sum test, and the significance (*P* < 0.05) was denoted by different superscripts

Similar to the cecum, *C. perfringens* (F6) and several pathobionts such as *Escherichia/Shigella* (F13), *E. faecium* (F27), and *E. cecorum* (F52) tended to increase or were significantly enriched, while dietary treatments had a tendency to reduce their abundance in the ileum of NE-infected chickens (Fig. 9A). However, supplementation with 1.5 mg/kg DCA/butyrate appeared to further increase many of these pathobionts. SCFA-producing bacteria responded differently to dietary treatments. *Cuneatibacter* (F4) and *R. endophytica* (F118) were largely abolished by NE and remained minimal by all dietary treatments, while *Faecalibacterium* (F7 and F8) tended to be suppressed by NE, stayed diminished in response to butyrate or DCA, but were restored in the 0.75 mg/kg DCA/butyrate group and significantly elevated in the 1.5 mg/kg DCA/butyrate group (Fig. 9B). Although unresponsive to NE infection, *Bacteroides* (F5 and F9) remained minimal in the ileum and were only enriched in response to butyrate/DCA combination, particularly butyrate/1.5 mg/kg DCA (Fig. 9B).

Lactobacilli are reduced in the ileum of NE-infected chickens proportional to the disease severity [20]. In this study with chickens of varying degrees of NE severity, group A *Lactobacillus* (F1) exhibited a tendency to decrease in the NE-infected ileum, but were largely restored to healthy levels in response to dietary treatments (Fig. 9C). No obvious NE-induced changes were observed with *L. salivarius* (F2), group B *Lactobacillus* (F3), *L. pontis* (F55), or *L. animalis* (F115), although two most effective treatments (1.5 mg/kg DCA and 0.75% DCA/butyrate) tended to enrich *L. pontis* and *L. animalis* (Fig. 9C). Another Lactobacillaceae family member, *Weissella paramesenteroides* (F56), tended to be reduced by NE and remained diminished in response to dietary treatments (Fig. 9C).

## Discussion

Modulation of endogenous HDP synthesis is being actively explored for disease control and prevention [38–42]. Supplementation of small-molecule HDP inducers or their combinations has shown efficacy in the alleviation of diseases [38–42]. Both butyrate and DCA are capable of inducing HDP synthesis separately [10–15]. In this study, we have shown a strong synergy in inducing the expression of multiple HDP genes between butyrate and DCA in both chicken macrophage cells and jejunal explants. Both compounds further synergize with each other in augmenting the expression of claudin-1*,* a major tight junction protein gene. Importantly, we show a combination of butyrate and DCA to be synergistic in protecting chickens from NE in two independent animal trials, suggesting its potential as an antibiotic alternative for mitigation of NE and possibly other enteric diseases.

Dietary supplementation of 1.5 g/kg DCA was found earlier to be highly protective against NE in broiler chickens [16–18], which is consistent with our results. In this study, we show a concentration-dependent alleviation of NE when supplementing 0.5, 1.0, and 1.5 g/kg DCA, with 1.5 g/kg giving the best outcome. Although it shows no obvious adverse effect on feed intake or weight gain in our 17-d trials, 1.5 g/kg DCA could negatively impact the long-term growth performance or physiology of healthy animals. For example, dietary supplementation of 1.0 g/kg of chenodeoxycholic acid, a less toxic primary bile acid, for two weeks significantly reduced feed intake and body weights of broiler chickens [43], while 0.6 g/kg chenodeoxycholic acid in the diet had a negligible impact on growth performance of 21-day-old weanling piglets [44]. Therefore, it is beneficial to reduce DCA in the diet while protecting animals. In our study, 0.75 g/kg DCA in combination with 1 g/kg NaB achieves the same efficacy as 1.5 g/kg DCA in NE alleviation in two independent animal trials. Further research is needed to explore the influence of the combination of 0.75 g/kg DCA and NaB on health and growth performance of chickens. The efficacy of using lower concentrations of DCA and NaB for protecting against NE warrants further investigation as well.

Synergistic induction of HDP and claudin-1 gene expression by DCA and butyrate is expected to contribute to enhanced innate mucosal defense, barrier function, and disease resistance. However, the mechanisms by which DCA and butyrate cooperate to upregulate HDP and claudin-1 expression remain to be explored. DCA and other bile acids are known to bind to several receptors such as farnesoid X receptor (FXR), Takeda G protein-coupled receptor 5 (TGR5), and vitamin D receptor (VDR) to exert their physiologic functions [8, 45]. Some of these receptors has been confirmed to be critical for HDP induction in human cells [12, 15]. Butyrate, on the other hand, is known to enhance the expression of VDR [46] and FXR [47], which are expected to enhance DCA signaling. Therefore, it is not surprising to observe a synergy between butyrate and DCA. However, experimental verification is required to validate participation of a particular bile acid receptor in the cooperation between butyrate and DCA on HDP and claudin-1 induction.

Additionally, the extent to which augmented HDP and tight junction protein syntheses contribute to NE alleviation remains uncertain. In fact, DCA and butyrate are each capable of modulating a myriad of host metabolic and immune responses [8, 48]. Moreover, DCA was found earlier to have direct antibacterial activity, with 50 μmol/L suppressing *C. perfringens* growth by 83% in bacterial culture [18]. However, the antibacterial activity of DCA could not fully explain the protective effect of DCA, because supplementation of 0.8 and 1.5 mg/kg DCA could lead to an accumulation of DCA to approximately 1 and 5 mmol/L in the ileum, respectively [17]. Both are well above the antibacterial concentration, yet only 1.5 mg/kg DCA provides significant protection against NE. Therefore, it is plausible that many of these activities of DCA and butyrate cooperate to achieve significant NE alleviation in live animals.

NE causes drastic changes to the intestinal microbiota. We have observed an obvious enrichment of *C. perfringens* and pathobionts such as *Escherichia/Shigella* and *Enterococcus* in both the cecum and ileum of NE-infected chickens, along with a concomitant diminishment of SCFA-producing bacteria such as *Faecalibacterium, Bacteroides,* and *Cuneatibacter*, as reported in earlier studies [20, 49]. The two most effective dietary treatments, i.e., 1.5 g/kg DCA and 0.75 g/kg DCA/butyrate, are capable of restoring the balance and composition of the intestinal microbiota to a large extent, but not fully to healthy levels. This is perhaps not surprising, given the fact that these interventions are not 100% effective. However, it is also possible that these dietary treatments protect the host without relying on complete restoration of the intestinal microbiota. For example, relative to healthy chickens, *Escherichia/Shigella* remains significantly elevated, while *Cureatibacter* is diminished in both NE-infected cecum and ileum in response to the combination of 0.75 g/kg DCA and butyrate. It remains unknown whether the alterations in the microbiota induced by DCA and butyrate are the cause or the consequence of their protective mechanism against NE.

Group A *Lactobacillus* consists of highly related *L. acidophilus, L. crispatus*, *L. amylovorus,* and *L. gallinarum*, while Group B *Lactobacillus* is comprised of *L. gasseri* and *L. johnsonii* [50]. Although sequencing the V3–V4 region of bacterial 16S rRNA gene is unable to distinguish between Group A and B *Lactobacillus*, it does provide a sufficient resolution to separate different genera of lactic acid bacteria from each other. We have detected *L. salivarius, L. reuteri, L. pontis*, and *L. animalis* and found that they are differentially regulated by NE and dietary interventions. Lactobacilli are gradually abolished in the ileum of the chickens proportional to the severity of NE infection [20]. In this study, a reduction of lactobacilli is not obvious in NE-infected ileum likely due to no separation of chickens with various NE severities. However, to our surprise, all major *Lactobacillus* species including Group A and Group B *Lactobacillus* and *L. salivarius* are drastically enriched in the cecum of NE chickens. While present below 3% in healthy cecum, *L. salivarius* becomes the most dominant, accounting for 22% of total bacteria in the cecum of NE-infected chickens. Group A *Lactobacillus* also shows a significant increase, rising from less than 3% in the healthy cecum to over 7% in the NE-infected cecum, where it also becomes a dominant bacterium. Consistently, *L. crispatus* (a Group A member) and *L. salivarius* were reported to be drastically increased in the cecum in response to NE, but *L. johnsonii* (a Group B member) was decreased [49].

While lactobacilli are reduced in the ileum of chickens, these bacteria are enriched in the cecum in response to NE. This shift is likely indicative of significant environmental changes in different segments of the intestinal tract, driven by the proliferation of *C. perfringens*, which produces toxins, SCFAs, and various metabolites, leading to intestinal inflammation and alterations in intestinal pH [51]. Interestingly, a similar enrichment of lactobacilli and bifidobacteria is observed in the colon of patients with inflammatory bowel disease [52]. Although most *Lactobacillus* species are considered probiotic, they can become opportunistic pathogens in immunocompromised individuals [53]. Therefore, the role of lactobacilli in the progression of NE remains unclear. Further research is warranted on whether lactobacilli actively contribute to NE development or simply adapt to the pro-inflammatory conditions of the gut. It is worth noting that the effective dietary treatments have largely restored lactobacilli to healthy levels.

Intestinal microbiota produces a diverse repertoire of metabolites with essential roles in host metabolism and immune defense [54, 55]. SCFAs and secondary bile acids are two major classes of microbiota-derived metabolites [54, 55]. However, how these metabolites cooperate with each other in exerting benefits to the host remains largely unexplored. Here we present an example of synergistic HDP induction and host defense by SCFAs and secondary bile acids. Apparently, cooperative actions in regulating host metabolic and immune responses among different microbiota-derived metabolites are likely to occur in vivo*.* It is important to further explore a potential synergy in different physiological functions among various metabolites.

## Conclusion

In summary, we have demonstrated a synergy between DCA and butyrate in inducing the expression of HDPs and claudin-1 both in vitro and ex vivo. The synergy is further confirmed in vivo, where we observe a significant improvement in animal survival, body weight gain, and intestinal pathology in animals supplemented with a combination of DCA and butyrate in a chicken model of NE. While SCFA-producing bacteria such as *Bacteroides, Faecalibacterium,* and *Cuneatibacter* are greatly diminished, and lactobacilli become the most dominant species in the cecum of NE-infected chickens, DCA and butyrate supplementation largely restores the intestinal microbiota to healthy levels. Taken together, these results highlight the potential use of DCA in combination with butyrate as an antibiotic alternative. It will be important to evaluate the efficacy of intestinal microbiota-derived metabolites, such as DCA and butyrate in growth promotion and disease prevention in poultry and livestock.

### Supplementary Information

**Additional file 1: Fig. S1.** Concentration-dependent alleviation of necrotic enteritis (NE) by deoxycholic acid (DCA). **Fig. S2.** Synergy between DCA and butyrate in alleviating necrotic enteritis (NE) in broiler chickens. **Table S1.** Pairwise comparisons of β-diversity of the d 17 cecal microbiota among different groups. **Table S2. **Pairwise comparisons of β-diversity of the d 17 ileal microbiota among different groups.

## Data Availability

The raw sequencing reads of this study have been deposited in the NCBI Sequence Read Archive (SRA) database under BioProject PRJNA725022.

## Abbreviations

ASV: Amplicon sequence variant
AvBD: Avian β-defensin
DCA: Deoxycholic acid
FTG: Fluid thioglycollate
FXR: Farnesoid X receptor
GAPDH: Glyceraldehyde 3-phosephate dehydrogenase
HDP: Host defense peptide
LDA: Linear discriminant analysis
LEfSe: Linear discriminant analysis effect size
NaB: Sodium butyrate
NE: Necrotic enteritis
RT-qPCR: Reverse transcription-quantitative PCR
SCFA: Short-chain fatty acid
SEM: Standard errors of the mean
TGR5: Takeda G protein-coupled receptor 5
VDR: Vitamin D receptor
