# Supplementary material for: Two intestinal microbiota-derived metabolites, deoxycholic acid and butyrate, synergize to enhance host defense peptide synthesis and alleviate necrotic enteritis
Source: J Anim Sci Biotechnol. 2024 Mar 2;15:29. doi: 10.1186/s40104-024-00995-9 (PMC10908072; doi:10.1186/s40104-024-00995-9)
Supplement: Supplementary file 1 — Additional file 1: Fig. S1. Concentration-dependent alleviation of necrotic enteritis (NE) by deoxycholic acid (DCA). Fig. S2. Synergy between DCA and butyrate in alleviating necrotic enteritis (NE) in broiler chickens. Table S1. Pairwise comparisons of β-diversity of the d 17 cecal microbiota among different groups. Table S2. Pairwise comparisons of β-diversity of the d 17 ileal microbiota among different groups. [file 40104_2024_995_MOESM1_ESM.docx]

**Supplementary Information**

**Additional file 1**

**Fig. S1.** Concentration-dependent alleviation of necrotic enteritis (NE) by deoxycholic acid (DCA). A total of 150 day-of-hatch male Cobb broilers were allotted to one of five groups (*n* = 30) supplemented with or without 0.5, 0.75, or 1.5 g/kg DCA. Four groups of animals were subjected to NE by sequential infections with *Eimeria maxima* on d 10 and *Clostridium perfringens* on d 14, while the remaining one group of 30 chickens were mock-infected as negative controls. **A** Animal survival (%) between d 14–17. ^**^*P* < 0.01, ^***^*P* < 0.001, and ^****^*P* < 0.0001 (relative to the NE group) based on the log-rank test. **B** Individual body weight gains of surviving animals between d 10–17. Data shown are mean ± SEM. Means not sharing a common superscript letter denote statistical significance (*P* < 0.05) based on one-way ANOVA and *post hoc* Tukey’s test. (**C**) Frequency (%) of jejunal lesion scores on d 17


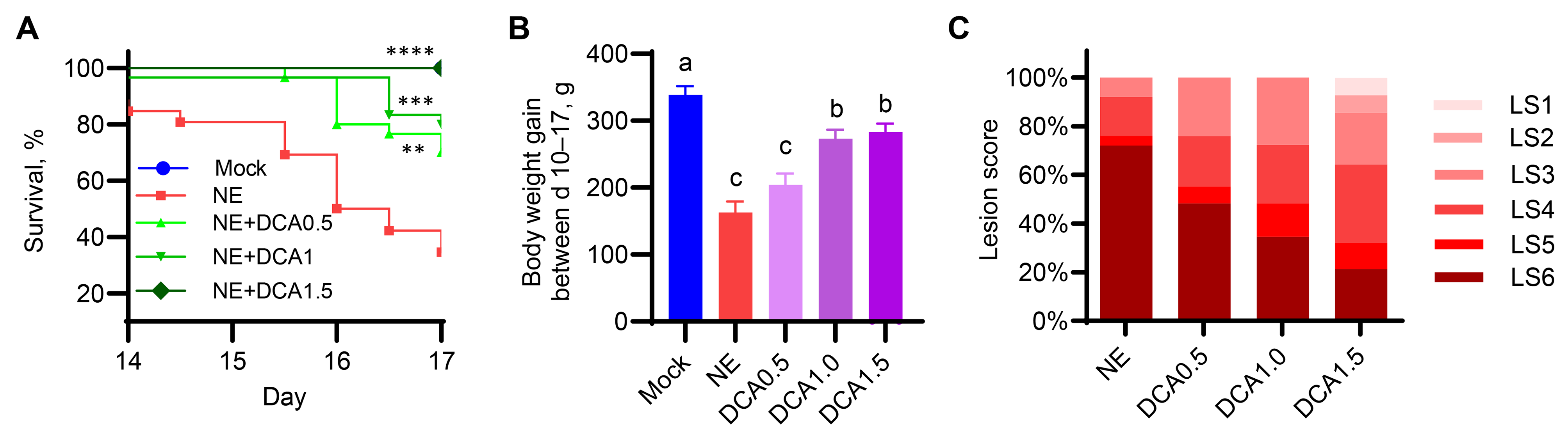

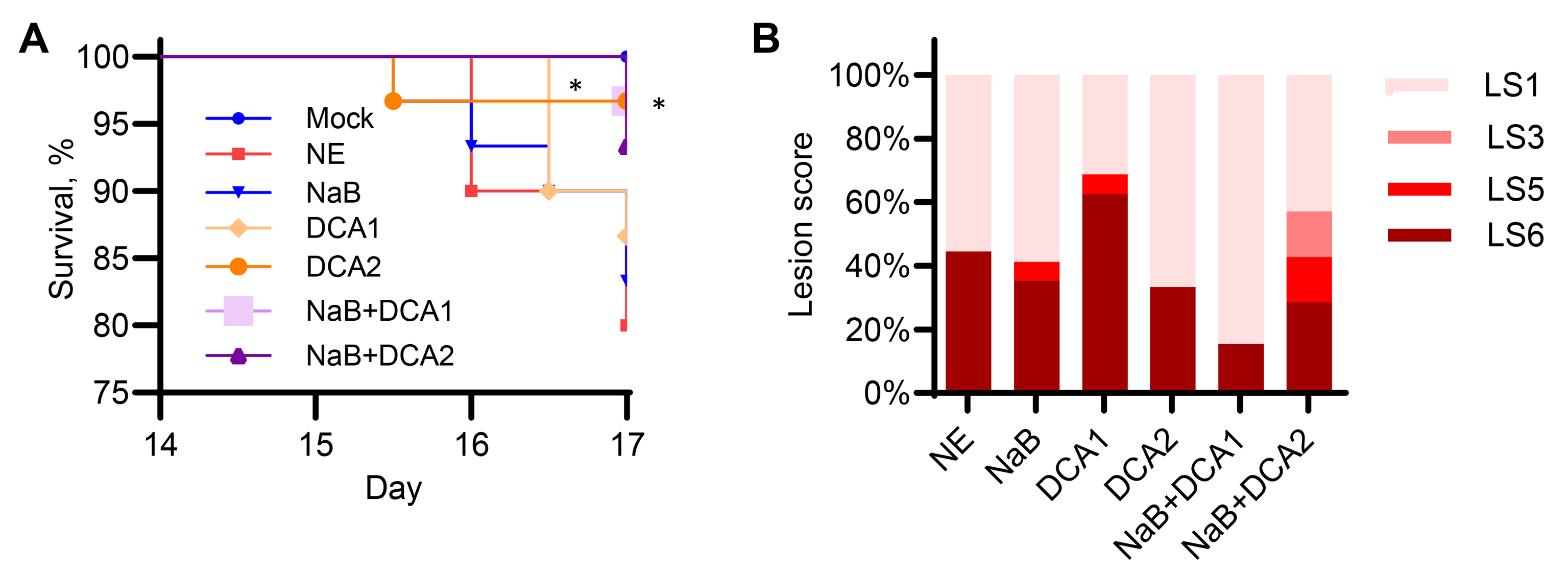


**Fig. S2.** Synergy between DCA and butyrate in alleviating necrotic enteritis (NE) in broiler chickens. A total of 210 day-of-hatch male Cobb broilers were allotted to one of seven groups (*n* = 30) supplemented with or without 1 g/kg NaB, 0.75 g/kg DCA (DCA1), 1.5 g/kg DCA (DCA2), or a mixture of 1 g/kg NaB and 0.75 g/kg DCA (NaB + DCA1) or 1 g/kg NaB and 1.5 g/kg DCA (NaB + DCA2). Six groups of animals were subjected to NE by sequential infections with *Eimeria maxima* on d 10 and *Clostridium perfringens* on d 14, while the remaining one group of 30 chickens were mock-infected as negative controls. **A** Animal survival (%) between d 14–17. Note that two groups (DCA2 and NaB + DCA1) are significantly different from the NE group (^*^*P* < 0.05) based on the log-rank test. **B** Frequency (%) of jejunal lesion scores on d 17

**Table S1** Pairwise comparisons of β-diversity of the d 17 cecal microbiota among different groups

| **Group** | **Mock** | **NE** | **NaB** | **DCA1** | **DCA2** | **NaB+DCA1** | **NaB+DCA2** |
| --- | --- | --- | --- | --- | --- | --- | --- |
| Mock |  | 0.001  (0.191) | 0.001  (0.129) | 0.001  (0.263) | 0.001  (0.312) | 0.001  (0.258) | 0.001  (0.206) |
| NE | 0.001  (0.329) |  | 0.001  (0.163) | 0.001  (0.304) | 0.001  (0.312) | 0.001  (0.208) | 0.001  (0.210) |
| B | 0.012  (0.150) | 0.001  (0.230) |  | 0.001  (0.228) | 0.001  (0.245) | 0.001  (0.169) | 0.001  (0.164) |
| DCA1 | 0.094  (0.102) | 0.001  (0.269) | 0.086  (0.105) |  | 0.001  (0.167) | 0.001  (0.233) | 0.001  (0.194) |
| DCA2 | 0.004  (0.277) | 0.001  (0.289) | 0.001  (0.200) | 0.414  (0.044) |  | 0.001  (0.215) | 0.005  (0.158) |
| B+DCA1 | 0.001  (0.263) | 0.008  (0.167) | 0.004  (0.147) | 0.039  (0.123) | 0.032  (0.113) |  | 0.012  (0.108) |
| B+DCA2 | 0.003  (0.275) | 0.001  (0.263) | 0.008  (0.177) | 0.121  (0.087) | 0.328  (0.058) | 0.43  (0.046) |  |

**Note:** Day-of-hatch male Cobb broilers were supplemented with or without 1 g/kg NaB, 0.75 g/kg DCA (DCA1), 1.5 g/kg DCA (DCA2), or a mixture of 1 g/kg NaB and 0.75 g/kg DCA (NaB + DCA1) or 1 g/kg NaB and 1.5 g/kg DCA (NaB + DCA2). Six groups of animals were subjected to NE, while the remaining group was mock-infected. The cecal digesta were randomly collected from 12 surviving animals/group on d 17 and subjected to 16S rRNA gene sequencing. *P*-values and *R*^2^ (in parentheses) of pairwise comparisons of weighted (grey shaded) and unweighted (blue shaded) UniFrac distances of different groups were determined by PERMANOVA using 999 permutations

**Table S2** Pairwise comparisons of β-diversity of the d 17 ileal microbiota among different groups

| **Group** | **Con** | **NE** | **NaB** | **DCA1** | **DCA2** | **NaB+DCA1** | **NaB+DCA2** |
| --- | --- | --- | --- | --- | --- | --- | --- |
| Con |  | 0.003  (0.185) | 0.002  (0.229) | 0.002  (0.267) | 0.002  (0.219) | 0.001  (0.226) | 0.001  (0.309) |
| NE | 0.094  (0.117) |  | 0.473  (0.067) | 0.006  (0.142) | 0.011  (0.174) | 0.035  (0.139) | 0.001  (0.243) |
| B | 0.002  (0.379) | 0.004  (0.212) |  | 0.207  (0.092) | 0.022  (0.145) | 0.199  (0.101) | 0.002  (0.231) |
| DCA1 | 0.005  (0.247) | 0.349  (0.074) | 0.010  (0.261) |  | 0.004  (0.211) | 0.003  (0.181) | 0.001  (0.342) |
| DCA2 | 0.023  (0.176) | 0.195  (0.094) | 0.002  (0.180) | 0.395  (0.064) |  | 0.113  (0.099) | 0.002  (0.235) |
| B+DCA1 | 0.004  (0.281) | 0.021  (0.142) | 0.087  (0.149) | 0.201  (0.099) | 0.276  (0.074) |  | 0.034  (0.109) |
| B+DCA2 | 0.031  (0.143) | 0.355  (0.062) | 0.018  (0.193) | 0.123  (0.098) | 0.361  (0.054) | 0.247  (0.078) |  |

**Note:** Day-of-hatch male Cobb broilers were supplemented with or without 1 g/kg NaB, 0.75 g/kg DCA (DCA1), 1.5 g/kg DCA (DCA2), or a mixture of 1 g/kg NaB and 0.75 g/kg DCA (NaB + DCA1) or 1 g/kg NaB and 1.5 g/kg DCA (NaB + DCA2). Six groups of animals were subjected to NE, while the remaining group was mock-infected. The proximal ideal digesta were randomly collected from 12 surviving animals/group on d 17 and subjected to 16S rRNA gene sequencing. *P*-values and *R*^2^ (in parentheses) of pairwise comparisons of weighted (grey shaded) and unweighted (blue shaded) UniFrac distances of different groups were determined by PERMANOVA using 999 permutations
